# Eslicarbazepine acetate: its effectiveness as adjunctive therapy in clinical trials and open studies

**DOI:** 10.1007/s00415-016-8338-2

**Published:** 2017-01-18

**Authors:** S. D. Shorvon, E. Trinka, B. J. Steinhoff, M. Holtkamp, V. Villanueva, J. Peltola, E. Ben-Menachem

**Affiliations:** 1 UCL Institute of Neurology, Box 5, National Hospital for Neurology and Neurosurgery, Queen Square, London, WC1N 3BG UK; 2 Department of Neurology and Neuroscience Institute at Christian Doppler Klinik, Paracelsus Medical University Salzburg, Ignaz Harrerstrasse 79, 5020 Salzburg, Austria; 3 Epilepsiezentrum Kork, Landstraße 1, 77694 Kehl-Kork, Germany; 4 Epilepsy-Center Berlin-Brandenburg, Department of Neurology, Charité-Universitätsmedizin Berlin, Charitéplatz 1, 10117 Berlin, Germany; 5 Multidisciplinary Epilepsy Unit, Neurology Service, Hospital Universitario y Polotécnico La Fe, Avda Fernando Abril Martorell 106, 46026 Valencia, Spain; 6 Department of Neurology, Tampere University Hospital, PO Box 2000, 33521 Tampere, Finland; 7 Institute of Neuroscience and Physiology, Sahlgrenska Academy, University of Göteborg, Box 430, SE-405 30 Göteborg, Sweden; 8 Centre for Cognitive Neuroscience, 5020 Salzburg, Austria

**Keywords:** Adjunctive therapy, Antiepileptic drug, Clinical practice, Epilepsy, Eslicarbazepine acetate, Focal-onset seizures

## Abstract

Eslicarbazepine acetate (ESL) is a once-daily antiepileptic drug that is approved as adjunctive therapy in adults with focal-onset seizures. Following oral administration, ESL is rapidly metabolized to its active metabolite, eslicarbazepine, which acts primarily by enhancing slow inactivation of voltage-gated sodium channels. The efficacy and safety/tolerability of ESL in the adjunctive setting were established in a comprehensive Phase III program (*n* = 1702 randomized patients) and this evidence has been supported by several open studies (*n* = 864). ESL treatment has demonstrated improvements in health-related quality of life, in both randomized clinical trials and open studies. ESL has also been shown to be usually well tolerated and efficacious when used in the adjunctive setting in elderly patients. The effectiveness of ESL as the only add-on to antiepileptic drug monotherapy has been demonstrated in a multinational study (*n* = 219), subgroup analyses of which have also shown it to be efficacious and generally well tolerated in patients who had previously not responded to carbamazepine therapy. Open studies have also demonstrated improvements in tolerability in patients switched overnight from oxcarbazepine to ESL. Due to differences in pharmacokinetics, pharmacodynamics, and metabolism, there may be clinical situations in which it is appropriate to consider switching patients from oxcarbazepine or carbamazepine to ESL.

## Introduction

Eslicarbazepine acetate (ESL) is a once-daily (OD) antiepileptic drug (AED) that is approved in Europe as adjunctive therapy in adults with focal-onset seizures, with or without secondary generalization [1], and, in the USA, for the treatment of focal-onset seizures as monotherapy or adjunctive therapy [2]. The efficacy and safety/tolerability of ESL as adjunctive therapy for focal-onset seizures in adults have been established in several randomized, double-blind, placebo-controlled, Phase III trials [3–6] and long-term extension studies [7–9]. In addition, an open-label, non-controlled Phase III trial has assessed the safety and efficacy of adjunctive ESL treatment in elderly patients (aged ≥65 years) [10].

Controlled clinical trials are essential in the licensing process of a new AED, but they typically employ strict inclusion/exclusion criteria and rigid dosing and titration schedules; whereas, in everyday clinical practice, patients are more diverse in terms of clinical characteristics, such as age, comorbidities and comedications, and treatment is individualized to each patient’s needs [11, 12]. ‘Real-world’ open studies are, therefore, required to complement evidence from clinical trials, by determining how the efficacy of an agent translates into effectiveness in clinical practice and by providing pragmatic guidance on optimal dosing and titration schedules. An aspect of determining the effectiveness of a treatment is to assess its impact on patients’ quality of life (QoL). This is particularly important for chronic conditions, such as epilepsy, where the effectiveness of treatment relies first and foremost on the patient’s willingness and ability to be compliant with the treatment over the long term. Since its approval, ESL’s safety and effectiveness have been investigated in open, unblinded studies [13–17], and its effects on QoL have been investigated in both the clinical trial setting and open studies [7, 8, 13, 18].

The aims of this article were to provide a brief overview of ESL’s pharmacology and to review current clinical evidence for ESL as an adjunctive treatment for adults with focal-onset seizures, from the Phase III clinical trials and some substantial open, unblinded studies.

## ESL pharmacology

Following oral administration, ESL is rapidly and extensively metabolized by first-pass hepatic hydrolysis to eslicarbazepine (S-licarbazepine), the active metabolite responsible for its pharmacological effect [19]. Eslicarbazepine accounts for approximately 94% of plasma drug exposure following oral administration of ESL, other moieties being R-licarbazepine (~5%) and oxcarbazepine (OXC; <1%) [20]. Eslicarbazepine displays linear pharmacokinetics at clinically relevant ESL doses and its effective half-life is 20–24 h [21].

**Fig. 1:**
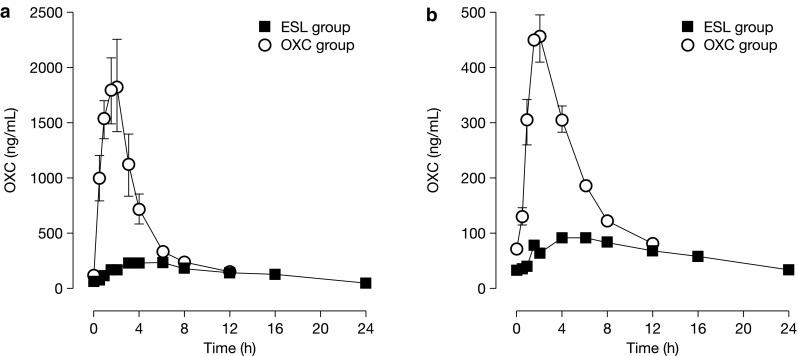
Plasma (**a**) and CSF (**b**) concentration–time profiles of OXC following the last dose of a repeated-dose regimen of once-daily ESL 1200 mg and twice-daily OXC 600 mg to healthy volunteers (*n* = 7 in each group for plasma profile; *n* = 6 in each group for CSF profile). *CSF* cerebrospinal fluid, *ESL* eslicarbazepine acetate, *OXC* oxcarbazepine. Adapted from Nunes et al. [20] with permission from John Wiley and Sons

ESL is a member of the dibenzazepine family of AEDs, which also includes OXC and carbamazepine (CBZ) [22]. ESL shares with OXC and CBZ the dibenzazepine nucleus bearing the 5-carboxamide substitute, but is structurally different from these agents at the 10,11-position [21, 23], resulting in differences in pharmacokinetics, pharmacodynamics, and metabolism [24]. Whereas ESL is stereoselectively metabolized primarily to eslicarbazepine, OXC is metabolized to both eslicarbazepine and R-licarbazepine, as well as being detectable in serum as the parent compound [20]. Although exposure to eslicarbazepine, assessed as area under the time–concentration curve (AUC), is similar following administration of OXC 600 mg twice daily and ESL 1200 mg OD (OXC/ESL ratio for AUC: 97% for plasma and 112% for cerebrospinal fluid [CSF]), exposure to R-licarbazepine is higher for OXC than for ESL (OXC/ESL ratio for AUC: 417% for plasma and 407% for CSF), as is exposure to oxcarbazepine (OXC/ESL ratio for AUC: 411% for plasma and 327% for CSF) [20]. ESL’s stereoselective metabolism, therefore, avoids the early peak in OXC concentration observed in plasma and CSF following immediate-release OXC administration (Fig. 1), which correlates with OXC-related adverse events (AEs; e.g., dizziness, headache) [20]. This difference may explain the finding from clinical trials that ESL is associated with fewer neurological AEs than immediate-release OXC [25]. Moreover, a retrospective, single-center study of 21 patients demonstrated that patients switched overnight from immediate-release OXC to ESL showed improved tolerability, as assessed using the Adverse Events Profile questionnaire [26]. ESL also potentially differs from CBZ in terms of its tolerability profile, since CBZ metabolism is associated with the generation of toxic metabolites, whereas ESL metabolism is not [27, 28]. Furthermore, CBZ is a potent enzyme inducer, reducing the duration and action of many drugs [29], and this contributes to the development of comorbidities such as osteoporosis, sexual dysfunction, and vascular disease [29, 30]. Eslicarbazepine, the main active metabolite of ESL after oral administration in humans, is a weak inducer of cytochrome P450 3A4 and uridine 5′-diphospho-glucuronosyl transferases [1], but it is a less potent enzyme inducer than CBZ. It should be noted that, since eslicarbazepine decreases exposure to the oral contraceptives, levonorgestrel and ethinylestradiol, most likely due to induction of cytochrome P450 3A4, women of childbearing potential should use adequate contraception during ESL treatment and up to the end of the current menstruation cycle after treatment has been discontinued [1].

It is thought that eslicarbazepine acts primarily by reducing the availability of voltage-gated sodium channels (VGSCs) through enhancement of slow inactivation [31], and, therefore, differs from CBZ, which acts by altering the fast inactivation of VGSCs [31]. Eslicarbazepine’s apparent affinity for VGSCs in the inactivated state is approximately two-fold less than that of CBZ [32] and its apparent affinity for VGSCs in the resting state is 5- to 15-fold lower than those of CBZ, OXC, and (R)-licarbazepine [19]. Eslicarbazepine, therefore, appears to have enhanced inhibitory selectivity for rapidly firing ‘epileptic’ neurons over those with normal activity [19, 33, 34]. The clinical significance of these differences is currently not known, but they could potentially play a role in the observed efficacy of ESL in the presence of CBZ resistance [35, 36]. Experiments using patch-clamp recording in human and rat hippocampal slices have demonstrated that eslicarbazepine exhibits maintained use-dependent blocking effects, with significant add-on effects to CBZ in human epilepsy [36]. These findings are supported by ESL clinical trial data demonstrating that ESL may be effective in patients whose seizures are uncontrolled by CBZ [3–6, 37]. Eslicarbazepine also differs from CBZ and R-licarbazepine in its effects on Ca_V_3.2 inward currents, sub-maximal GABA currents, K_V_7.2 outward currents, and glycine GlyRα3 receptor-mediated inward currents [34]. Although the potential clinical significance of these differences is also not yet known, ESL has been shown to exhibit strong antiepileptogenic effects in experimental models of epilepsy that may in part be due to its inhibitory effects on Ca_V_3.2 T-type Ca^2+^ channels [36].

## ESL Phase III clinical trial data

### Data from randomized, double-blind, placebo-controlled, Phase III trials

The efficacy and safety/tolerability of ESL as adjunctive therapy in adults with focal-onset seizures have been investigated in four international, multicenter, Phase III trials: Studies 301 [3], 302 [4], 303 [5], and 304 [6]. All of these individual Phase III trials met their primary endpoints. The results of a post hoc analysis of pooled data from the randomized, double-blind, placebo-controlled periods of Studies 301, 302, and 303 will be presented here [38]. Subsequent findings from Study 304 [6] and a post hoc pooled analysis of Studies 301, 302, and 304 [39–41] are consistent with the findings of the pooled analysis of Studies 301, 302, and 303 outlined below. ESL was licensed as an adjunctive treatment for focal-onset seizures by the European Medicines Agency on the basis of Studies 301, 302, and 303 [1], and by the United States Food and Drug Administration on the basis of Studies 301, 302, and 304 [2].

#### Summary of pooled analysis of studies 301, 302, and 303

The trials included patients aged ≥18 years with a documented diagnosis of epilepsy and at least a 12-month history of simple or complex focal-onset seizures, with or without secondary generalization [38]. Patients were also required to be treated with stable doses of one or two AEDs (one to three AEDs in Study 302). The predefined key efficacy endpoints for the pooled analysis were seizure frequency during the 12-week maintenance period (adjusted per 4 weeks), relative reduction from baseline in seizure frequency, and responder rate (response defined as ≥50% seizure frequency reduction from baseline). These endpoints were assessed for the intention-to-treat (ITT) and per protocol (PP) populations. Safety assessments included treatment-emergent AEs (TEAEs), clinical laboratory parameters, vital signs, and electrocardiography (ECG) [38].

**Fig. 2:**
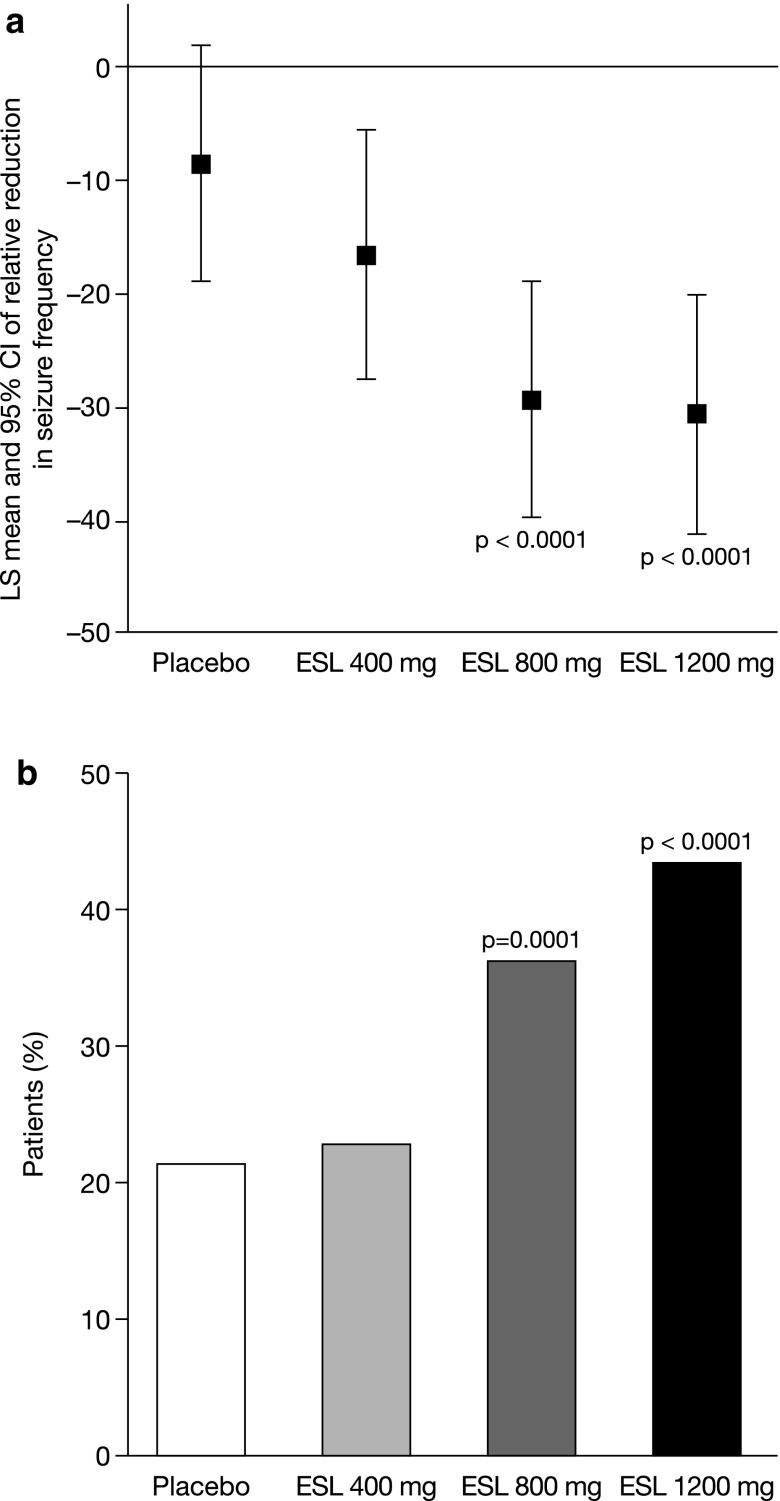
Efficacy analysis of pooled data from Phase III Studies 301, 302, and 303: **a** relative reduction from baseline in seizure frequency during 12-week maintenance treatment with adjunctive ESL (ITT population) and **b** responder rate during 12-week maintenance treatment with adjunctive ESL (ITT population) [38] reprinted with permission from John Wiley and Sons. Response was defined as ≥50% reduction from baseline in seizure frequency; *p*-values refer to comparison vs. placebo. *CI* confidence interval, *ESL* eslicarbazepine acetate, *ITT* intention-to-treat, *LS* least squares

Data obtained from 1049 patients enrolled at 125 centers in 23 countries were pooled and analyzed [38]. The majority of the population was Caucasian and approximately 50% of patients were males. The mean age was approximately 37 years and the mean duration of epilepsy was 22 years. Compared with placebo, there was a statistically significant reduction in seizure frequency during the maintenance period with ESL 800 mg/day and ESL 1200 mg/day in both the ITT and PP populations (*p* < 0.0001; Fig. 2a). The median relative reduction in seizure frequency was 35% with ESL 800 mg/day and 39% with ESL 1200 mg/day, compared with 15% with placebo (ITT population). Similarly, the responder rate was significantly higher for ESL 800 mg/day (36%) and ESL 1200 mg/day (44%), compared with placebo (22%) (*p* = 0.0001 and *p* < 0.0001, respectively; ITT population; Fig. 2b) [38].

The incidence of TEAEs was higher for ESL than for placebo and increased with ESL dose (Table 1) [38]. The majority of TEAEs were of mild or moderate intensity. The most frequently reported TEAEs (≥10% of patients in any treatment group) were dizziness, somnolence, headache, and nausea (Table 1). Differences in the frequencies of TEAEs between the ESL and placebo groups were mainly observed during the first 6 weeks of treatment, after which the frequencies across groups were similar. TEAEs leading to discontinuation were also dose-related (Table 1); these were mainly vertigo, diplopia, blurred vision, nausea and vomiting, fatigue, abnormal coordination, dizziness, headache, and somnolence. No dose-dependent trend was observed for serious TEAEs. Only one patient died during the study (placebo group). Changes in mean clinical laboratory parameters did not yield clinically relevant findings and there were no changes in vital signs or body weight of clinical concern. Hyponatremia <125 mM was reported in four patients [ESL 400 mg/day, *n* = 1 (0.5%); ESL 800 mg/day, *n* = 2 (0.7%); ESL 1200 mg/day, *n* = 1 (0.4%)]. All four patients were concomitantly treated with CBZ at ≥1000 mg/day and all had sodium levels <135 mM at baseline. ESL treatment was associated with no clinically relevant ECG findings. No clinically significant prolongation of the QTc interval was observed in any patient [38].

**Table 1:** Summary of TEAEs in pooled analysis of Studies 301, 302, and 303 [38]

	Placebo (*n* = 289)	ESL 400 mg/day (*n* = 196)	ESL 800 mg/day (*n* = 284)	ESL 1200 mg/day (*n* = 280)	Total ESL (*n* = 760)
Any TEAE, *n* (%)	134 (46.4)	119 (60.7)	178 (62.7)	189 (67.5)	486 (63.9)
TEAEs with incidence ≥10% in any treatment group, *n* (%)					
Dizziness	21 (7.3)	26 (13.3)	60 (21.1)	81 (28.9)	167 (22.0)
Somnolence	27 (9.3)	21 (10.7)	37 (13.0)	42 (15.0)	100 (13.2)
Headache	25 (8.7)	17 (8.7)	29 (10.2)	38 (13.6)	84 (11.1)
Nausea	6 (2.1)	10 (5.1)	21 (7.4)	28 (10.0)	59 (7.8)
TEAEs considered possibly related to ESL treatment, *n* (%)	72 (24.9)	75 (38.3)	134 (47.2)	154 (55.0)	363 (47.8)
TEAEs by severity, *n* (%)					
Mild	56 (19.4)	56 (28.6)	72 (25.4)	56 (20.0)	184 (24.2)
Moderate	65 (22.5)	45 (23.0)	82 (28.9)	101 (36.1)	228 (30.0)
Severe	13 (4.5)	18 (9.2)	24 (8.5)	32 (11.4)	74 (9.7)
TEAEs leading to discontinuation, *n* (%)	13 (4.5)	17 (8.7)	33 (11.6)	54 (19.3)	104 (13.7)
Any serious TEAE, *n* (%)	4 (1.4)	9 (4.6)	10 (3.5)	9 (3.2)	28 (3.7)
Deaths, *n* (%)	1 (0.3)	0	0	0	0

*ESL* eslicarbazepine acetate, *TEAE* treatment-emergent adverse event

#### Influence of starting dose and dose titration scheme on incidence of TEAEs

The influence of starting dose and dose titration scheme on the incidence of TEAEs during treatment with ESL was examined as part of the post hoc pooled analysis of Studies 301, 302, and 304 [41]. During the 2-week titration period, there was a marked difference between the TEAE profile of the ESL 800 mg/day ‘without-titration’ group and the ESL 800 mg/day ‘with-titration’ group, the incidence of all of the most frequently reported TEAEs being higher without titration than with. The greatest differences were for dizziness (24.9 vs. 8.5%), somnolence (15.9 vs. 5.5%), headache (10.8 vs. 4.5%), nausea (12.2 vs. 3.0%), vomiting (7.3 vs. 1.0%), and ataxia (6.1 vs. 0.5%). Among the treatment groups with a target dose of ESL 800 or 1200 mg/day, the frequency of the most commonly reported TEAEs was higher for those initiated at 800 mg/day versus 400 mg/day. The frequency of TEAEs in the ESL 800 mg/day ‘with-titration’ and ESL 1200 mg/day ‘with-titration’ groups was similar to the ESL 400 mg/day group, and not markedly different from placebo. The incidence of rash did not appear to be related to ESL starting dose or to the rate of dose escalation, but was higher among patients maintained on ESL 1200 mg/day (2.6–4.9%) than among patients maintained on ESL 800 mg/day (0.0–1.9%), ESL 400 mg/day (0.5%), or placebo (0.9%) [41].

Overall, these Phase III randomized, controlled trial data demonstrated that ESL was effective and well tolerated as an adjunctive therapy for adults with focal-onset seizures [3–6, 38–41]. ESL led to dose-related improvements in most efficacy outcomes, the effective dose range being 800–1200 mg OD [38]. The overall incidence of TEAEs was higher at higher doses of ESL, which appears attributable to expected AEs, such as diplopia, dizziness, headache, vertigo, and somnolence. The incidence of serious TEAEs in these studies was low. TEAEs were generally predictable, manageable, occurred during the early stages of treatment, and were of mild to moderate intensity [38]. The pooled analysis of Studies 301, 302, and 304 also indicated that the frequency of TEAEs with ESL may be minimized by use of an appropriate titration scheme [41].

### Data from open-label Phase III studies

#### ESL safety/tolerability and efficacy in elderly patients

The safety/tolerability and efficacy of adjunctive ESL therapy in elderly patients (aged ≥65 years) with focal-onset seizures were assessed in a multicenter, open-label, non-controlled, single-arm Phase III trial [10]. The trial employed flexible doses of ESL (400–1200 mg OD), in accordance with the recommendations approved by the regulatory authority [1]. Patients were included if they had at least two focal-onset seizures during the 8-week baseline period and were being treated with one or two AEDs other than OXC. After an 8-week baseline period, patients entered a 26-week maintenance period, during which ESL was initiated at 400 mg OD and adjusted based on individual response (400–1200 mg/day). Safety/tolerability was assessed by evaluation of TEAEs, clinical laboratory evaluations, vital signs, 12-lead ECG, physical/neurological examinations, Norris’ scales for evaluation of sedative effects, and the Columbia Suicide Severity Rating Scale. Efficacy was assessed as the absolute and relative change from baseline in seizure frequency (standardized to frequency per 4 weeks), responder rate (response defined as ≥50% seizure frequency reduction), and seizure freedom rate [10].

The study population comprised 72 patients (52.8% males), with a mean age of 71.6 years (range 65–84 years) [10]. The mean treatment duration was 151.8 days and the mean ESL dose during the overall treatment period was 591.9 mg/day. The majority of patients received doses no higher than 800 mg/day. The most frequently reported TEAEs (≥5% patients) were dizziness (12.5%), somnolence (9.7%), fatigue (8.3%), convulsion (8.3%), hyponatremia (8.3%), nasopharyngitis (6.9%), and upper respiratory tract infection (5.6%). The majority of TEAEs were of mild or moderate intensity. In total, 16 serious TEAEs were reported for ten (13.9%) patients; none occurred in more than one patient. Three patients died but none of the deaths was considered related to study medication. Sixteen (22.2%) patients discontinued due to TEAEs. TEAEs leading to discontinuation of more than one patient were hyponatremia (*n* = 3), dizziness (*n* = 2), and fatigue (*n* = 2). Laboratory-related TEAEs affecting more than one patient were hyponatremia (8.3%), increased blood creatine phosphokinase (4.2%), and increased gamma-glutamyltransferase (4.2%). For vital signs, ECG, and physical and neurological examinations, no trends were observed and the incidence of relevant findings was low. The Norris’ adapted mental sedation scales showed minor changes in patient responses towards a slight worsening of mean values. There were no reports of suicidality post baseline, as assessed by the Columbia Suicide Severity Rating Scale and TEAE reporting [10].

For the full analysis set (*n* = 71), the responder and seizure freedom rates during the maintenance period were 54.9 and 15.5%, respectively [10]. The corresponding values for the PP set (*n* = 55) were 56.4 and 12.7%, respectively. The mean (standard deviation) standardized seizure frequency decreased from 4.8 (5.5) during the 8-week baseline period to 3.6 (5.8) during the 26-week maintenance period (full analysis set). Overall, the study found that adjunctive treatment with ESL (400–1200 mg OD) in elderly patients with focal-onset seizures was efficacious and did not raise any unexpected safety concerns [10].

#### Impact of ESL on QoL

During the 1-year, open-label extension studies of the Phase III adjunctive therapy trials, patients were treated with flexible ESL dosing (400–1200 mg/day) according to response and tolerability [7–9]. These studies included an assessment of the long-term impact of adjunctive ESL treatment on health-related QoL, by employing the Quality of Life in Epilepsy Inventory-31 (QOLIE-31) questionnaire [42] at baseline of the initial Phase III trial and at the end of 1-year open-label treatment or at early discontinuation [7, 8, 18]. In the open-label extension of Study 301, QOLIE-31 scores increased (i.e., improved) from baseline to the last assessment and the improvement was statistically significant for all subscales except emotional well-being [7]. The mean relative improvement in QOLIE-31 subscale scores ranged from 7.1 (emotional well-being) to 51.4 (seizure worry), and the overall mean score improved from 54.8 at baseline to 58.3 at the last assessment (*p* < 0.0001) [7]. Similar improvements in QOLIE-31 scores were observed in the open-label extensions of Study 302 (significant improvements in the overall QoL, seizure worry, and medication effects subscales and overall score) [8] and Study 303 (significant improvements in all subscales and overall score) [18].

### Phase IV open study data

#### EPOS study

The Eslicarbazepine acetate in Partial-Onset Seizures (EPOS) study was a prospective, non-interventional, open-label investigation conducted in 88 sites across eight European countries [13]. Its objectives were to assess the retention rate, seizure control, safety/tolerability and effect on QoL of ESL as add-on to antiepileptic monotherapy in everyday clinical practice. Adult patients with focal-onset seizures (with or without secondary generalization), insufficiently controlled under AED monotherapy, were offered participation in the study if their clinician had previously and independently decided to initiate ESL add-on therapy. ESL was recommended to be used according to approved guidance [1]. The primary endpoint was retention rate after 6 months. Other assessments included retention rate after 3 months, and efficacy, safety/tolerability, and QoL after 3 and 6 months. Efficacy was assessed as seizure frequency during the previous 3 months, responder rate (response defined as ≥50% seizure frequency reduction from baseline), and seizure freedom rate (seizure freedom defined as no seizures within the previous 3 months). Safety/tolerability was assessed by evaluating AEs and adverse drug reactions, defined as AEs with causal relationship to study drug. QoL was assessed using the patient-rated Quality of Life in Epilepsy Inventory-10 (QOLIE-10) [13, 43].

**Fig. 3:**
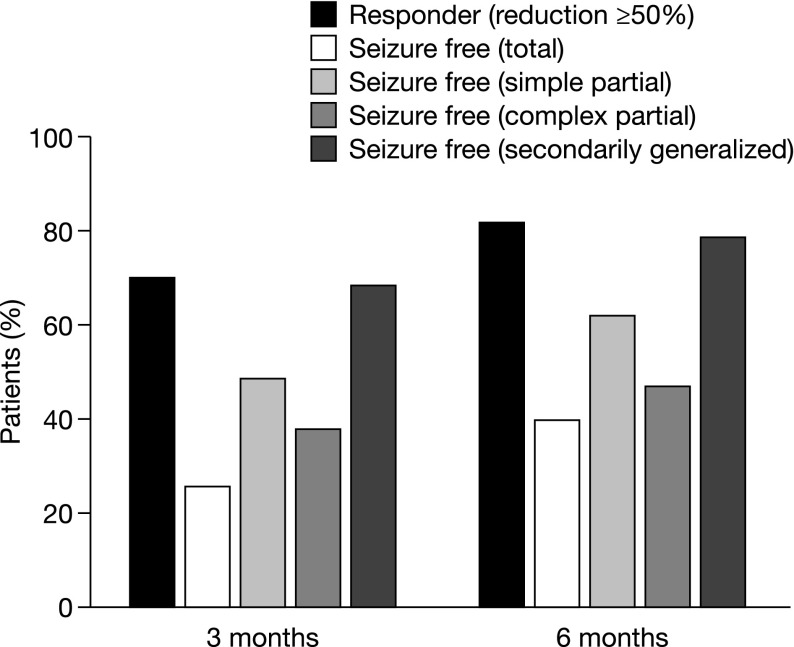
Responder and seizure freedom rates after 3 and 6 months in the EPOS study. Response was defined as ≥50% seizure frequency reduction in the previous 3 months, compared with the 3 months prior to initiating ESL therapy. Seizure freedom is presented for total seizures and by seizure type. *n* = 212 at 3 months; *n* = 189 at 6 months [13] reprinted with permission from John Wiley and Sons

A total of 219 patients were included in the study [13]. The median age was 43 years (range 18–83 years) and 57.5% were males. The mean time since epilepsy diagnosis was 12.3 years (range 0–57.3 years). Most patients (74.3%) received a target ESL dose of 800 mg/day. For the majority of patients (79.3%), the target dose was reached with one titration step. The most commonly used baseline AED monotherapies (≥5% of patients) were levetiracetam (37.9%), lamotrigine (24.7%), valproate (13.7%), and carbamazepine (6.4%). The 6-month retention rate was 82.2% [95% confidence interval (CI) 76.5–87.0%] and the 3-month retention rate was 89.0% (95% CI: 84.1–92.9%). After 3 and 6 months, responder rates were 69.9 and 81.8%, respectively, and seizure freedom rates were 25.9 and 39.2%, respectively (Fig. 3). AEs were reported for 26.0% of patients and adverse drug reactions for 22.4% of patients. Eight patients (3.7%) experienced serious AEs. No AE was reported for >5% of patients. The most frequently reported AEs were dizziness (4.6%), headache (3.2%), convulsion (3.2%), and fatigue (2.7%). The mean QOLIE-10 score decreased (i.e., improved) from 2.9 (*n* = 128) at baseline to 2.4 (*n* = 114) after 3 months and 2.1 (*n* = 109) after 6 months [13].

Post hoc subgroup analyses were conducted for those 45 patients in EPOS who had documented non-response to historic CBZ treatment [44] and the 41 patients aged >60 years [45]. Efficacy, safety/tolerability, and the impact of treatment on QoL were assessed as for the overall population [13]. In the subgroup of patients who had previously not responded to CBZ treatment, the retention, responder, and seizure freedom rates after 6 months were 88.9% (95% CI: 75.9–96.3%), 95.1% (95% CI: 83.5–99.4%), and 33.3% (95% CI: 19.6–49.5%), respectively, and the mean QOLIE-10 score decreased from 2.8 (*n* = 21) at baseline to 2.2 (−13.0%; *n* = 18) after 6 months [44]. Two AEs were reported for two (4.4%) patients and both were hyponatremia [44]. Similarly, for the elderly patients included in EPOS, the retention, responder, and seizure freedom rates after 6 months were 78.0% (95% CI: 62.4–89.4%), 83.3% (95% CI: 65.3–94.4%), and 56.3% (95% CI: 37.7–73.6%), respectively, and the mean QOLIE-10 score decreased from 2.7 (*n* = 28) at baseline to 2.2 (−14.5%; *n* = 24) after 6 months [45]. Twelve AEs were reported for six (14.6%) patients and no AE was reported in >5% of patients. The most frequently reported AEs were dizziness (4.9%) and allergic dermatitis (4.9%) [45].

Overall, the EPOS study demonstrated that ESL as add-on to antiepileptic monotherapy was associated with favorable retention and seizure control, and was well tolerated by the majority of adult patients [13]. ESL treatment also resulted in improvements in patient-rated QoL [13] in patients who were less severely ill at baseline than those enrolled in clinical trials. Moreover, ESL was shown to be effective and generally well tolerated when used in elderly patients, and in those who had previously not responded to CBZ therapy [44, 45].

#### Spanish ESLIBASE study

The ESLIBASE study was a multicenter, retrospective, non-interventional study undertaken to evaluate the long-term efficacy and safety of adjunctive ESL therapy in patients with focal epilepsy in a clinical practice setting [14]. Conducted in 12 hospitals in Spain, the study included patients with a diagnosis of epilepsy and focal-onset seizures who were treated with ESL according to clinical practice and whose ESL treatment was initiated between January 2010 and July 2012. Data were collected retrospectively at baseline and at 3, 6, and 12 months. Efficacy assessments included responder rate (response defined as ≥50% seizure frequency reduction from baseline), seizure freedom rate, and retention rate, all assessed after 3, 6, and 12 months. Safety assessments included evaluation of AEs [14].

The study population comprised 327 patients (52.0% female) with a mean age of 41.9 years (range 14–87 years) [14]. For most patients, ESL was initiated at 400 mg/day as a single dose and up-titrated in 400-mg increments every 7, 10, or 14 days until the optimal dose was reached. The maximal approved dose (1200 mg/day) was exceeded if deemed necessary; 26 (7.9%) patients were taking doses >1200 mg/day at last observation. The median ESL dose at Months 3, 6, and 12 was 800, 1200, and 1200 mg/day, respectively (Villanueva, personal communication), with doses ranging from 400 to 2000 mg/day at every timepoint. There was a significant decrease in mean number of concomitant AEDs used from baseline (2.0) to last follow-up (1.6; *p* < 0.001) [14].

**Fig. 4:**
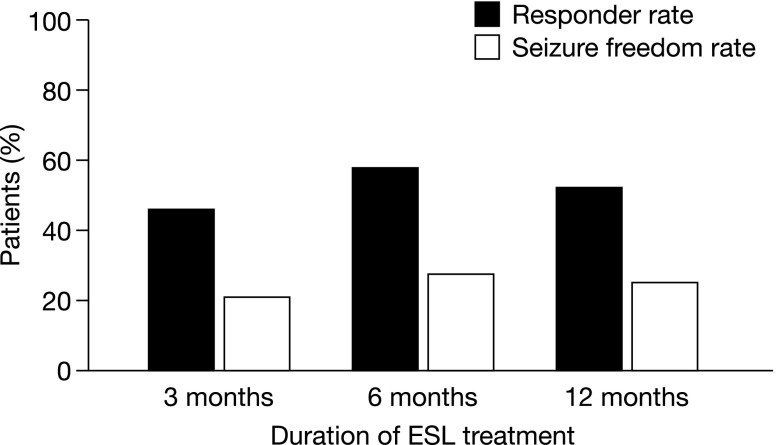
Responder and seizure freedom rates after 3, 6, and 12 months of ESL treatment in the ESLIBASE study (*n* = 327). Response was defined as ≥50% seizure frequency reduction from baseline. Seizure freedom was defined as no seizures from the beginning of the study (up to timepoints earlier than the 12-month visit), no seizures for the last 6 months (at the 12-month visit), or no seizures for 12 months if patients were seizure free in the 3 months prior to study entry [14] reprinted with permission from Elsevier

Retention rates after 3, 6, and 12 months were 89.3, 80.1, and 72.5%, respectively [14]. After 12 months, 52.5% of patients were responders and 25.3% of patients were seizure free (Fig. 4). The cumulative rate of AEs that were possibly related to ESL treatment was 40.7% at 12 months. The most commonly reported TEAEs (≥5% of patients) were dizziness/nausea (11.3%), somnolence (6.1%), and ataxia (5.1%). Rash/pruritus was reported for 12 (3.6%) patients and hyponatremia (ranging from 116–128 mEq/L) was reported for nine (2.7%) patients. The majority of AEs were mild or moderate in intensity. The cumulative rate of AEs leading to treatment discontinuation was 16.2% after 12 months. Of 26 patients who were transitioned from OXC to ESL due to OXC-related AEs, 15 (57.7%) no longer had AEs after transitioning to ESL. Similarly, of 17 patients who were transitioned from CBZ to ESL due to CBZ-related AEs, eight (47.1%) no longer had AEs after transitioning to ESL [14].

#### Other open studies

A retrospective, consecutive, 2-year observational study assessed the efficacy and tolerability of adjunctive ESL therapy in 152 patients (mean age 38.5 years; eight patients <18 years) treated at a single center in Portugal [15]. Patients’ mean epilepsy duration was 26.8 years and their mean seizure frequency in the 3 months prior to ESL initiation was 19.7 seizures/month. Kaplan–Meier retention rates were 82.9, 71.3, 65.1, and 62.8% at 6, 12, 18, and 24 months, respectively. Retention was shown to be unaffected by gender, diagnosis, age, or epilepsy duration. Overall, 56 patients (36.8%) discontinued ESL treatment: 32 (57.1%) due to AEs, 19 (33.9%) due to lack of efficacy, and five (8.9%) due to other reasons. Responder rates at 6, 12, 18, and 24 months were 25.7, 25.7, 19.0, and 17.1%, respectively. AEs were reported by 64 patients (42.1%), half of whom discontinued ESL therapy due to AEs. The most frequently reported AEs were dizziness and somnolence/slowness. AEs were more frequently reported in treatment regimens that included CBZ. Overall, no new safety signals emerged compared with evidence from ESL clinical trials [15].

An observational, descriptive, cross-sectional study was conducted to assess efficacy and tolerability in the first 61 patients to receive adjunctive ESL therapy for drug-resistant epilepsy at a single epilepsy unit in Spain [16]. The mean follow-up duration was 4.7 ± 3.2 months and the retention rate at 3 months was 75.4%. In 40 patients with a minimum follow-up period of 3 months, monthly median seizure frequency decreased from baseline by 63.6% (*p* < 0.001); 12 patients (30.0%) achieved a reduction of ≥80%, and five (12.5%) achieved seizure freedom. AEs were reported by 35 patients (57.4%) and mostly occurred during titration. The most commonly reported AE was dizziness (34.4%). Two patients experienced exanthematic cutaneous reactions and four patients (6.6%) developed hyponatremia (sodium range 128–132 mmol/L). There were no sodium values <125 mmol/L and no patients discontinued ESL due to low sodium levels. Twelve patients (19.7%) switched overnight to ESL from OXC, using a dose ratio of 1:1, and 13 patients (21.3%) switched overnight from CBZ to ESL, using a dose ratio of 1:1.3. Switching from OXC to ESL was found to be effective and well tolerated, whereas switching from CBZ to ESL was less effective and less well tolerated [16].

In another audit of 105 patients treated with ESL at a single unit in Spain, 20.7% of patients remained seizure-free and 58.4% demonstrated >50% seizure frequency reduction after the introduction of ESL [17]. After 6 months, 18.1% of patients had experienced AEs (the most common being cognitive disorders) and 11.5% had discontinued treatment. The addition of ESL to lacosamide was shown to be significantly less effective in controlling seizures than its addition to other AEDs, whereas the addition of ESL to other sodium channel blockers was shown to be similar in efficacy to its addition to other AEDs [17].

## Discussion

The clinical efficacy and safety/tolerability of adjunctive ESL therapy in adults with focal-onset seizures have been established in a program of randomized, double-blind, placebo-controlled, Phase III trials [3–6]. These trials have also established the effectiveness of adjunctive ESL therapy by including patient-reported outcome measures that have demonstrated improvements in health-related QoL over the long term [7, 8, 18]. ESL has additionally been shown to be well tolerated and efficacious when used in the adjunctive setting in elderly patients with focal-onset seizures [10].

ESL is approved in Europe at doses up to 1200 mg/day as adjunctive therapy in adults with focal-onset seizures [1]. Since ESL is additionally approved in the USA at doses up to 1600 mg/day as monotherapy in adults with uncontrolled focal-onset seizures [2], on the basis of the findings of two Phase III conversion to monotherapy trials [46, 47], it remains to be determined whether the 1600-mg/day dose might also be effective and well tolerated in the adjunctive setting.

There has been increasing acknowledgement of the importance of open studies in helping to inform health policy decisions, and bodies such as the International Society for Pharmacoeconomics and Outcomes Research have highlighted the need for rigor and transparency when conducting such studies [48, 49]. In the case of ESL, clinical trial data have been supported by several open studies, which have demonstrated that adjunctive ESL treatment was effective and generally well tolerated as long-term therapy in clinical practice [14, 15], and when used as the only add-on to AED monotherapy in clinical practice [13]. The effectiveness of adjunctive ESL treatment in clinical practice was associated with favorable retention and improvements in health-related QoL [13, 14]. Moreover, when used as the only add-on to AED monotherapy, ESL was shown to be efficacious and generally well tolerated in elderly patients [45] and in patients who had previously not responded to CBZ therapy [44].

Hyponatremia and rash have been reported as common AEs in patients treated with ESL in clinical trials (1.2 and 1.1%, respectively) [1]. Higher rates of hyponatremia have been reported in elderly patients [10] and in some post-marketing open studies [14, 16]. It is, therefore, good practice to monitor for the potential development of hyponatremia with ESL treatment through laboratory testing, particularly in the elderly. Rash has also been reported in some open studies [14, 16]. Neuropsychiatric and cognitive side effects are reported uncommonly with ESL treatment (≥1/1000 to <1/100 patients), with the exception of disturbance in attention (≥1/100 to <1/10 patients) [1].

The effectiveness of ESL in patients who are resistant to CBZ therapy may be due to differences between the agents in terms of their modes of action [31, 36]. Moreover, given the pharmacological differences between ESL and OXC and CBZ [21, 23, 24], there may be other clinical situations in which it is appropriate to consider transitioning patients from CBZ or OXC to ESL [50]. When transitioning patients from OXC to ESL, a dose ratio of 1:1 is recommended and it has been claimed that the change is possible to undertake in a single step, with no adjustment to comedication required [50]. The transitioning of patients from CBZ to ESL is less straightforward and should be carefully considered on a case-by-case basis, taking account of the patient’s clinical characteristics and comedications, which may require dose adjustment due to CBZ being a strong inducer of CYP enzymes [50]. In general, a CBZ:ESL dose ratio of 1:1.3 should be used and patients should be transitioned over a minimum period of 1–2 weeks [50] although longer periods of switching are often advised. In addition to patients who are resistant to CBZ therapy, clinical situations in which it might be appropriate to consider a transition to ESL include patients who experience OXC- or CBZ-related AEs (e.g., cognitive AEs) and those who experience, or are at risk of developing, metabolic problems resulting from CBZ induction of enzymes involved in endogenous metabolic pathways (e.g., hypercholesterolemia, osteoporosis, sexual dysfunction) [50]. It should be noted, however, that long-term follow-up studies are required to confirm whether ESL can reduce the risk of the types of long-term metabolic sequelae reported for CBZ or not. Other patients for whom it might be appropriate to consider transitioning from CBZ or OXC to ESL are those who are poorly compliant with two- or three-times daily dosing [50].
